# An eHealth Application in Head and Neck Cancer Survivorship Care: Health Care Professionals' Perspectives

**DOI:** 10.2196/jmir.4870

**Published:** 2015-10-21

**Authors:** Sanne Duman-Lubberding, Cornelia F van Uden-Kraan, Niels Peek, Pim Cuijpers, C René Leemans, Irma M Verdonck-de Leeuw

**Affiliations:** ^1^ Department of Otolaryngology / Head and Neck Surgery VU University Medical Center Amsterdam Netherlands; ^2^ Department of Clinical Psychology VU University Amsterdam Netherlands; ^3^ Health e-Research Centre University of Manchester Manchester United Kingdom

**Keywords:** cancer, tertiary prevention, participatory design approach, follow-up care, supportive care

## Abstract

**Background:**

Although many cancer survivors could benefit from supportive care, they often do not utilize such services. Previous studies have shown that patient-reported outcomes (PROs) could be a solution to meet cancer survivors’ needs, for example through an eHealth application that monitors quality of life and provides personalized advice and supportive care options. In order to develop an effective application that can successfully be implemented in current health care, it is important to include health care professionals in the development process.

**Objective:**

The aim of this study was to investigate health care professionals’ perspectives toward follow-up care and an eHealth application, OncoKompas, in follow-up cancer care that monitors quality of life via PROs, followed by automatically generated tailored feedback and personalized advice on supportive care.

**Methods:**

Health care professionals involved in head and neck cancer care (N=11) were interviewed on current follow-up care and the anticipated value of the proposed eHealth application (Step 1). A prototype of the eHealth application, OncoKompas, was developed (Step 2). Cognitive walkthroughs were conducted among health care professionals (N=21) to investigate perceived usability (Step 3). Interviews were recorded, transcribed verbatim, and analyzed by 2 coders.

**Results:**

Health care professionals indicated several barriers in current follow-up care including difficulties in detecting symptoms, patients’ perceived need for supportive care, and a lack of time to encourage survivors to obtain supportive care. Health care professionals expected the eHealth application to be of added value. The cognitive walkthroughs demonstrated that health care professionals emphasized the importance of tailoring care. They considered the navigation structure of OncoKompas to be complex. Health care professionals differed in their opinion toward the best strategy to implement the application in clinical practice but indicated that it should be incorporated in the HNC cancer care pathway to ensure all survivors would benefit.

**Conclusions:**

Health care professionals experienced several barriers in directing patients to supportive care. They were positive toward the development and implementation of an eHealth application and expected it could support survivors in obtaining supportive care tailored to their needs. The cognitive walkthroughs revealed several points for optimizing the application prototype and developing an efficient implementation strategy. Including health care professionals in an early phase of a participatory design approach is valuable in developing an eHealth application and an implementation strategy meeting stakeholders’ needs.

## Introduction

Many cancer survivors have to manage the adverse effects of cancer and its treatment. Head and neck cancer (HNC) specifically has an impact on survivors compared to other cancers. In addition to symptoms such as fatigue, HNC survivors are confronted with oral dysfunction, voice, speech, and swallowing problems, and related social withdrawal and psychological distress. These may negatively impact on quality of life (QOL) [1,2] and increase the need for supportive care.

Supportive care in cancer entails the prevention and management of the adverse effects of cancer and its treatment across the survivorship continuum [3,4]. Although many cancer survivors, including HNC survivors, could benefit from supportive care, they often do not utilize such services [5-8]. Barriers that stand in the way of obtaining supportive care include a lack of awareness of these services and a lack of identification of survivors’ symptoms and supportive care needs [9-11].

The use of patient-reported outcome measures (PROs) has been identified as a possible facilitator to detecting survivors’ symptoms [12]. Monitoring symptoms may be helpful in addressing survivors’ individual supportive care needs [13]. A prerequisite for its success is that monitoring should be followed by adequate referral to supportive care. An eHealth application integrating PROs to monitor QOL, followed by automatically generated tailored feedback and personalized advice on supportive care options, could be an alternative solution to meet cancer survivors’ individual needs. The proposed eHealth application could also be a helpful tool to enhance self-management among HNC survivors.

In a previous study, we investigated the attitude and preferences of cancer survivors toward an eHealth application targeting personalized referral to supportive care services [14]. The results of this needs assessment showed that survivors were indeed interested in this option of self-management support and believed that the eHealth application could eliminate barriers experienced in current follow-up care, for example, a minimal response from physicians concerning their needs and having to search for services themselves. The results also highlighted considerations and requirements concerning the application, for example, doubts about the degree of tailoring and the need for the application to be an addition to rather than a substitute for traditional care [14].

In order to develop an effective eHealth application and ensure adequate uptake, it is important to include all stakeholders, including health care professionals, during the entire developmental phase, following an iterative participatory approach [15]. Therefore, the main aim of this study was to investigate health care professionals’ perspectives toward an eHealth application in follow-up cancer care, which monitors QOL via PROs (Measure), followed by automatically generated tailored feedback (Learn), and personalized advice on supportive care (Act). The results of this study are intended to contribute to further development of a participatory design approach enabling the development of effective eHealth applications that meet stakeholders’ preferences and needs.

## Methods

**Figure 1:**
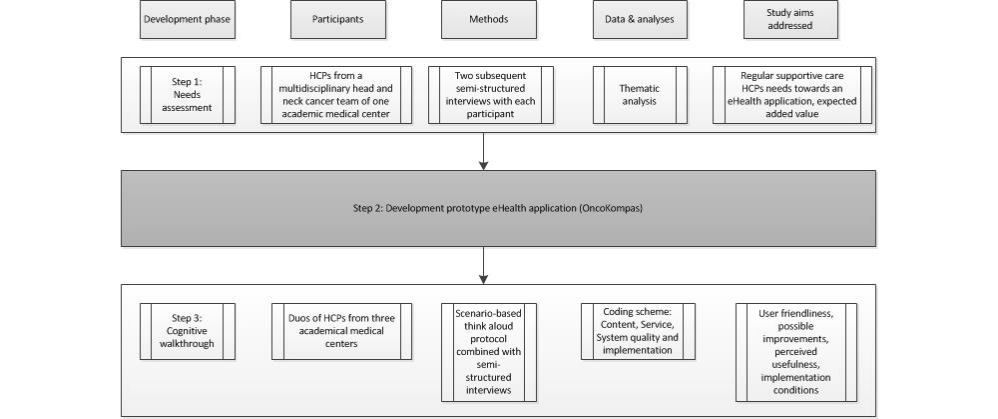
Study design.

A mixed methods study design was used consisting of 3 steps (Figure 1). We investigated health care professionals’ perspectives toward current follow-up care and toward the proposed eHealth application (Step 1) through a qualitative needs assessment. Next, we developed a prototype of the eHealth application (Step 2). Subsequently, we evaluated the application by means of cognitive walkthroughs (CWs) by health care professionals and investigated health care professionals’ opinions about usability and conditions for implementation (Step 3).

### Step 1: Needs Assessment

Health care professionals (N=11) were recruited from a multidisciplinary team involved in the care of HNC patients at the VU University Medical Center in Amsterdam, The Netherlands. We made use of purposive sampling. After permission from the department head, we requested study participation from a heterogeneous sample of health care professionals. The final sample consisted of an oral and maxillofacial surgeon, head and neck surgeon, oncologist, radiation oncologist, medical social worker, physiotherapist, dental hygienist, dietician, speech therapist, and 2 oncology nurses. Participating health care professionals’ experience in working with cancer patients ranged from 2 years and 3 months to 25 years (mean 13.38 years). Health care professionals were interviewed twice. An overview of the topics is shown in Table 1.

**Table 1:** Topics discussed in the needs assessment interviews.

Topic	Example question
Current follow-up care: assessing symptoms and supportive care needs	How do you assess patients’ symptoms and quality of life?
	What difficulties do you encounter when assessing patients’ symptoms and quality of life?
	How do you assess patients’ supportive care needs?
	Do you refer patients to supportive cancer care options?
	To which supportive care options do you refer patients?
	What difficulties do you encounter when referring patients to supportive cancer care options?
Added value of an eHealth tool in follow-up care for health care professionals	How may an eHealth application be supportive/fit into in your current role in follow-up cancer care?

The first interview covered questions about current follow-up care (assessing patient’s symptoms and need for supportive care). The second interview covered questions about the expected added value for health care professionals of an eHealth tool aimed at improving supportive care. In this second interview, more information about the proposed application was conveyed (eg, examples of personalized advice texts and supportive care options).

In total, 22 interviews were conducted, which lasted between 24 and 50 minutes (median 35, SD 7.24). All interviews were recorded and transcribed verbatim.

### Step 2: Development of the Prototype eHealth Application

A prototype of the eHealth application, “OncoKompas,” was developed based on the results of the needs assessment among health care professionals (from this study) and survivors [14]. Existing applications were used as examples to build the application [16,17]. First, the results of both needs assessments were discussed with the development team (Web designers and programmers), to translate these needs into requirements. The Web designer and programmers used their expertise to translate these requirements into a prototype of OncoKompas. During regular “demo sessions,” these requirements were revisited to ensure a proper translation into the prototype. The contents of OncoKompas were developed together with teams of experts consisting of cancer survivors, medical specialists, and paramedics (refer to the “Results” section for more details on OncoKompas).

### Step 3: Cognitive Walkthroughs

The cognitive walkthroughs (CWs) consisted of an expert-based usability evaluation followed by semistructured interviews. The health care professionals who participated in the needs assessment in Step 1 were complemented by a psychologist, a spiritual counsellor, and a patient advisor. We also included 3 head and neck surgeons, a radiation oncologist, 2 oncology nurses, and a health scientist from 2 other academic hospitals.

All but one of the usability evaluations were conducted in pairs of health care professionals because this was expected to increase “thinking out loud” by the participants. Health care professionals were asked to “walk through” the application guided by scenarios and user tasks from the end-users’ viewpoint. Following the usability evaluations, we interviewed the health care professionals on the implementation process (Table 2).

**Table 2:** Overview of CW scenario’s tasks and interview topics.

Scenario example	This scenario involves a 66-year-old female head and neck cancer patient. She is experiencing (the onset of) depression as well as stress at home. Furthermore, she has diarrhea and does not use a feeding tube or nutritional drinks. She has mild dysphagia and moderate loss of taste and smell.
CW tasks	Task 1: Monitor disease problems by filling out the PROs in OncoKompas and sending in the completed questionnaires.
	Task 2: View your personal well-being profile in OncoKompas.
	Task 3: Use personalized well-being profiles to find information regarding your physical condition related to your tumor.
	Task 4: Find personalized advice on an aspect of interest to you, and then take action based on this advice.
	Task 5: Find more information in OncoKompas regarding a particular supportive care option of your choice and then open and view the website of a recommended supportive care provider.
Semistructured interview topic: Implementation OncoKompas	What role do you think you could have in the usage of OncoKompas by patients?
	How do you think OncoKompas could be implemented in the regular follow-up care procedure?
	Do you intend to refer your patients to OncoKompas when available?

In total, 11 CWs were conducted, which lasted between 68 and 120 minutes (median 82, SD 14.54), and were recorded using Morae software (Morae version 2.1, TechSmith).

### Data Analysis

All needs assessment interviews and CWs were analyzed by thematic analysis [18]. Both coders (SDL and CvU) read all transcripts to familiarize themselves with the data. The coders independently selected citations from the transcripts of all needs assessment interviews relating to current follow-up cancer care and needs of health care professionals with respect to an eHealth application. These were coded into themes.

To analyze the usability of OncoKompas, we made use of the CW transcripts, supported by the Morae recordings. In total, 9 transcripts were coded by 2 coders. Initial codes for the CWs were generated focusing on system quality (ease of use), content quality (usefulness and relevance), and service quality (the process of care provided) [15,19,20]. Additionally, both coders independently selected citations for 9 of the semistructured interviews concerning the implementation process and coded these into categories. The remaining 2 CW transcripts were coded by 1 coder (SDL).

Next, the 2 coders met to review the extracted citations and themes from the needs assessment interviews and CWs. Disagreements were resolved through consensus, which was reached on all citations and themes. They developed 2 frameworks (one for the needs assessment and one for the CWs), in which the themes were identified and subthemes defined. After coding, the raw data were examined again to ensure the robustness of the analytical process and to ensure that all the data were reflected in the coding [21]. Quotations were translated from Dutch into English and anonymized.

## Results

### Step 1: Health Care Professionals’ Needs Assessment

#### Current Follow-Up Care: Assessing Symptoms and Supportive Care Needs

Health care professionals indicated that during consultations they typically ask the cancer survivors about their symptoms and undertake a physical examination. A few indicated they also asked their patients to complete PROs. Furthermore, when preparing for the consultations, health care professionals indicated that they consulted with their colleagues, as well as the electronic hospital information system (Table 3).

Health care professionals mentioned several difficulties in assessing survivors’ symptoms and in the referral process to supportive care services. They mentioned they are able to address only a limited scope of issues during the consultation due to limited time. In addition, all health care professionals said they tend to focus on their own field of expertise, for example, physicians indicated that they do not feel capable of assessing a survivor’s psychological well-being:

In an open setting you will of course ask: “Are there any things you’d like to discuss?” I think that works fine as a first move to also allow space for the psychological aspects, but of course you do ask things like: “How is your weight?,” “What about the pain?.”

Another difficulty according to health care professionals is that they do not want to burden the survivor with unnecessary questions about irrelevant or irreversible symptoms, for example, problems with salivary glands due to radiation therapy. In addition to this, they indicated that they lack a complete picture of survivors’ symptoms and quality of life. This comes about due to survivors’ hesitancy in mentioning all their symptoms and issues, as well as due to fragmentation in clinical care (eg, no insight into the patient information system of the other health care professionals involved).

**Table 3:** Overview of key issues and themes from the needs assessment.

Key issues			Themes
**Detecting symptoms and need for supportive care**			
	**Assessment of survivors’ symptoms**		
		Consulting survivor	Verbal questioning (based on checklist or according to protocol)
			Observing and physical examination (according to protocol)
			Wait and see what symptoms survivor describes
			Use of PROs (OncoQuest)
		Consulting colleagues	
		Consulting patient information system	
	**Barriers in determination**		
		Limited scope of issues being raised during consultation	Limited consultation time
			Limited skills or expertise of health care professional
			Limited responsibility of health care professional
		Do not wish to burden the survivor by asking about…	Irrelevant symptoms
			“Irreversible” symptoms caused by treatment
		No complete picture of a survivors’ symptoms	Patients do not mention all symptoms
			Fragmentation in care
**Current referral to supportive care options**			
	**Supportive care services referred**		
		Available services within the hospital	Allied health services, ie, physical therapist, dietician
		Services outside hospital	Specialized cancer centers
			Cancer rehabilitation program
			Allied health services in the region
			General practitioner
	**Barriers in referral**		
		Lack of options	Lack of overview of available and adequate supportive care
		Practical barriers in referral	Lack of time to encourage survivors to obtain supportive care
			Referral to region complicated due to lack of expertise on HNC
			Referral only possible through physician
		No need of survivor to be referred	Survivor is unwilling to be referred
			Survivor already has adequate supportive care
**Expected added value of eHealth application in follow-up care for health care professionals**			
	**Increases insight into symptoms**		
		Provides a complete picture of patients’ symptoms	Provides insight into the interdependence of patients’ symptoms
			Signal function: creates awareness of the severity of symptoms
			In support of their own observation/impression of health care professional
			By monitoring symptoms ability to serve as treatment outcome
		Improved (preparation for) consultation	Low threshold to speak up about specific issues/symptoms
			Option to target questions regarding specific symptoms
			Option to elaborate on and prioritize symptoms
	**Personalized advice/information**		
		Provides tailored information	More detailed information than provided by physician
			Back up for advice provided by health care professional
			Supportive to information provided by health care professional
		Platform to deliver additional care	Informative support to self-management advice
			Availability of physical therapy exercises
		Increases insight into QOL domains	Improved knowledge in QOL domains out of health care professionals’ expertise
	Insight into supportive care options		Increased insight into supportive care options
	Additional service in follow-up care		Showcase for hospital

Health care professionals indicated that care for HNC patients in The Netherlands is provided by multidisciplinary teams during treatment. However, follow-up care is generally provided only by physicians who continue to follow-up on the cancer survivor regularly. Physicians said they were hesitant to refer survivors to supportive care. In cases of mild symptoms, they provide the survivor with personal advice themselves. Where there are cases of severe symptoms, they refer survivors to other health care professionals. The supportive care services that health care professionals make their referrals to are often limited to other health care professionals in the same hospital. When referral takes place to services outside the hospital, these mainly include specialized centers for cancer survivors, cancer rehabilitation programs, allied health services in the region, or the survivor’s general practitioner.

Health care professionals also described barriers in referral to supportive care. They reported a lack of overview of the availability of supportive care services. Also, practical barriers in referral were mentioned, including a lack of time to encourage survivors to obtain supportive care:

What I usually do, is just say “this is available,” and if it will do some people good, they will give it a go if I want them to. In itself, that’s fine, but it is tricky, as you only have a short amount of time during a consultation. You have to encourage people too and that is often the problem.

Referral to allied health services in the region was considered complicated due to a lack of expertise in HNC. Finally, health care professionals indicated there was a perceived lack of need by the survivor to be referred, either due to unwillingness or due to the health care professionals’ assumption that the survivor already had adequate support.

#### Health Care Professionals’ Views on a Proposed eHealth Application in Follow-Up Cancer Care

Most health care professionals expected an eHealth application to provide added value for themselves in their practice, particularly in terms of follow-up care with the aim of optimizing supportive cancer care (Table 3). They hoped by using an eHealth application such as this to monitor survivors, to obtain an increased insight into these patients’ symptoms. In addition, the application could help detect survivors with severe symptoms. Health care professionals indicated that they anticipated the application could serve as a tool during their consultations, help prioritize symptoms, and support them in elaborating on and targeting questions toward symptoms.

Personalized information and advice for survivors provided by an eHealth application was expected to have an added value, if tailored to tumor type or treatment. Health care professionals indicated they expected this information to be supportive or supplementary to the information they provided to patients. Health care professionals expected that the application could also serve as a platform to deliver additional care, such as self-management advice and physical therapy exercises. Another benefit expected was an increased insight into various quality of life domains that were not part of the health care professionals’ specialty.

Insight into supportive care options available could be improved by means of an eHealth application. Finally, health care professionals expected the application to be an additional service for survivors in follow-up care, which could serve as a showcase for the hospital.

### Step 2: Prototype of OncoKompas

The prototype OncoKompas was developed in Step 2. OncoKompas was developed as an online computer application. It consists of the following 3 components: (1) Measure, (2) Learn, and (3) Act. In the “Measure” component, cancer survivors can independently complete PROs targeting the following QOL domains: physical functioning, psychological functioning, social functioning, healthy lifestyle, and existential issues*.* A specific domain containing topics for head and neck cancer patients is available, in addition to those general domains for cancer survivors (Table 4).

**Table 4:** Overview of OncoKompas topics*.*

Psychological QOL	Physical QOL	Social QOL	Healthy lifestyle	Existential issues	Head and neck cancer
Anxiety and depression	General everyday life	Social life	Alcohol	Life questions	Swallowing
Fear of recurrence	Pain	Relationship with partner	Physical activity	Religion	Speech
Subjective cognitive functioning	Sexuality	Relationship with children	Dietary intake	Future perspective	Oral function
Stress	Sleep quality	Financial circumstances	Weight		Neck and shoulder function
	Body image	Patient-physician communication	Smoking		Loss of smell and taste
	Fatigue	Return to work			Head and neck cancer specific lymphedema
	Diarrhea				Nutritional drink/Tube feeding
	Lack of appetite				
	Dyspnea				
	Nausea or vomiting				
	Constipation				
	Hearing and tinnitus				

On the basis of the interview results, specific PROs, validated questionnaires (or subscales) if available, were selected by the project team in collaboration with teams of experts. This selection was based on Dutch practice guidelines and literature searches. Data from the “Measure” component are processed in real-time and linked to tailored feedback to the cancer survivor in the “Learn” component. All algorithm calculations are based on available cutoff scores or are defined based on Dutch practice guidelines, literature searches, and/or consensus by teams of experts. A compass metaphor is used in the “Learn” component to summarize overall well-being. Once overall well-being has been presented, feedback is provided to the participant on the risk level for the topics (eg, depression, fatigue) by means of a 3-color system: green (no elevated well-being risks), orange (elevated well-being risks), and red (seriously elevated well-being risks). Cancer survivors receive elaborate personalized information on the outcomes. For instance, taking depression, information is provided on the symptoms of depression and the proportion of cancer survivors who suffer from depressive symptoms. Special attention is paid to evidence-based associations between outcomes. For example, feedback on the association between depression and fatigue is provided if a participant has an orange or a red score on depression as well as on fatigue. The feedback in the “Learn” component concludes with comprehensive self-care advice (tips and tools). All this advice is tailored to the individual cancer survivor, for example, tailored to age (eg, survivors over 70 years of age receive an adapted advice on exercising), gender (eg, advice on sexuality issues differ between men and women), and comorbidity (eg, dietary advice differs for diabetic patients).

In the “Act” component, survivors are provided with personalized supportive care options based on their PRO scores and expressed preferences (eg, preference for individual therapy versus group therapy). If a participant has elevated well-being risks (orange score), the feedback includes suggestions for self-help interventions. If a participant has “seriously elevated well-being risks” (red score), the feedback includes advice to contact their own medical specialist or general practitioner. If survivors want to share their results with their caregiver, they are able to “print their results to PDF” and either bring these with them (hard copy) during their consultation with the caregiver or email these results to the caregiver.

A clickable demo of the application (in Dutch) or an animation video (in Dutch and English) is available on the OncoKompas website.

### Step 3: Cognitive Walkthroughs

Technical errors occurred in 2 of the 11 CWs but were subsequently resolved. Health care professionals’ strengths and weaknesses concerning quality of the system, content, and service are presented in Multimedia Appendix 1.

#### System Quality

Health care professionals’ opinions toward the accessibility of OncoKompas varied. Many health care professionals indicated that OncoKompas may not be useful for a group of HNC survivors, due to limited eHealth literacy skills, lack of motivation, and older age. Others emphasized the usefulness of eHealth applications for HNC survivors, through the elimination of social barriers, such as difficulty speaking and shame about facial scarring. The 24/7 availability from home was considered important (see Multimedia Appendix 1).

According to the majority of health care professionals, the ease of use of OncoKompas was suboptimal because of the complicated navigation structure. Health care professionals mentioned that the interface was too busy for the target group. Complicating aspects included too much scrolling and unclear progress in the “Measure” component of OncoKompas. Positive aspects included a self-explanatory walkthrough of the application and the option to quit and save the questionnaire halfway through.

Health care professionals suggested the level of tailoring needed to be improved, for example, with respect to the advice provided. They considered the advice as distant and general, which could make it unclear to the survivor that the information had been tailored to their situation. Some mentioned that as participants are forced to monitor all symptoms, they might receive information on symptoms irrelevant to them. The provision of tailored advice, in contrast to surfing the Web was considered positive:

As I see it, the advantage of the program is that it makes the piles of available information accessible.

Finally, health care professionals suggested including reminders to encourage participants into action.

#### Content Quality

Health care professionals believed there was tension between the application goal and the use of evidence-based PROs. The use of evidence-based PROs requires participants to fill out more questions than needed to obtain personalized advice. However, the evidence-based content of the application was valued by health care professionals. The application mostly followed health care professionals’ own professional standards with respect to enquiring about symptoms and the provision of advice, which dovetailed with their advice to potential end users. In other words, the advice given in the application was the advice that health care professionals expected to be provided (see Multimedia Appendix 1):

When I look at it, it provides the advice that I would expect to be provided.

Health care professionals varied in their opinion regarding content comprehensibility. The “Measure” component was considered difficult by some health care professionals, as was the use of abstract terminology (eg, “well-being profile”). Others were positive about the different comprehensibility levels at which information was provided to participants. They complimented the formulation of advice texts and the different levels of information provided by the application (so-called read more options):

I believe that most people are able to gauge their own level pretty well. And people who cannot fully grasp this information, soon think, well, I have read all the tips, that will do.

The content was considered to be complete by most health care professionals. They were positive about the completeness of the QOL aspects included, their interdependence, and the diversity in supportive care options provided. Others indicated that the content was superfluous in that some information is provided to participants several times throughout OncoKompas. Some believed information was missing, for example, costs of supportive care options.

#### Service Quality of OncoKompas

Health care professionals were positive about the usefulness of OncoKompas in identifying symptoms, especially by providing patients with a complete picture of their well-being and insight into the interdependences, leading to a clarification of request for help:

OncoKompas is useful...by broadening the insight of patients and clarifying to them when the time has come to ask for help. Instead of having just us as health care professionals ask and explore, it can enable patients to become more pro-active in that respect.

Health care professionals’ concerns included that OncoKompas lacks nuance and may not be as tailored as a personal consultation with a health care professional (see Multimedia Appendix 1).

Health care professionals also indicated that they expected benefits in informing participants by creating an opportunity to receive information on sensitive topics. Others mentioned that survivors may be reluctant to use the application for information, because it is easier for them to contact the outpatient clinic. Some health care professionals expected that participants might receive inaccurate or irrelevant information if they inaccurately navigated through the application:

Well, with only a few wrong clicks, you can end up with the strangest of information. That does worry me a bit.

Health care professionals indicated that the application could support participants by referring them to appropriate supportive care options compatible with their symptoms. Some health care professionals mentioned concerns regarding whether participants would know what to do next. They expected participants to get lost in the supportive care options available to choose from, possibly leading to a lack of action.

Health care professionals also mentioned to expect some overall benefits for future participants, such as empowerment and increased engagement:

I can imagine this patient is wondering, “Do I have to bother my physician about that?” And when she receives the information from OncoKompas, she sees, “Yes, I should bother my physician about that.”

According to health care professionals, the application could also help participants be better prepared for their consultations. Concerns mentioned by health care professionals included an expected increase in workload and more consultations with health care professionals due to participants’ increased insight into whom to turn to with their symptoms. Some health care professionals mentioned that OncoKompas could possibly lead to participants’ continuing to obsess about their disease instead of helping them move on with their life or that emotions surrounding their cancer could (re)surface. Another negative consequence mentioned by health care professionals was that participants might not seek the expertise of a health care professional concerning their symptoms if they had already received information from the application.

#### Implementation of OncoKompas

Most health care professionals mentioned a positive intention to refer their patients to OncoKompas. All health care professional agreed that if the application were to be implemented in daily clinical practice, it should be offered to survivors through a routine procedure in a care pathway. Physicians believed that referral to the application should take place from different sources, including outside the hospital (eg, by the Dutch Cancer Society). Health care professionals suggested possibilities to increase awareness, such as providing a demo in the waiting room (see Multimedia Appendix 2).

Health care professionals differed in their opinion toward the best strategy to implement the application in clinical practice. Several health care professionals believed that OncoKompas should be implemented as a self-management instrument (independent use by survivors), while others stressed the use as a *supported* self-management instrument (with support from a health care professional).

Implementation as a self-management instrument was expected to stimulate survivor empowerment and to support survivors in defining their own route to relieving their symptoms and increasing their quality of life. Furthermore, health care professionals mentioned that survivors are responsible for their own well-being. Health care professionals indicated that referral of survivors to their physician by means of OncoKompas in case of severe symptoms would relieve them from the responsibility to take action on symptoms they may not know are present. Health care professionals argued that with a self-management application, survivors’ privacy would remain intact. They expected survivors to answer more truthfully if they knew their physician would not have access to the data:

When a patient wants to share their results, that would be nice, but I think the additional value also lies in that he has the opportunity to keep it to himself.

Finally, health care professionals expected that they could get around difficulties in discussing OncoKompas results during their regular consultations (eg, difficulties due to time pressure and the priority to check for cancer recurrence) by offering HNC survivors access to OncoKompas as an unsupported self-management application.

Other health care professionals indicated that OncoKompas should be implemented as a *supported* self-management tool because the responsibility of survivors’ well-being always remains with the health care professional. Health care professionals wished to receive feedback through access to OncoKompas or a system alert. They wanted to use the results to discuss these and prioritize symptoms during their consultations. Health care professionals indicated they were aware that when OncoKompas is implemented as a supported self-management tool, this requires action from the health care professionals in cases where survivors receive negative results from the application. Health care professionals mentioned that they might not always be able to fulfil this expectation, possibly leading to survivor disappointment:

It might be that it raises false expectations in the patient. As surely there will be times that I won’t come round to it and if the patient then expects, the doctor will have a quick read when I am there and we are going to discuss what I have filled in, then that is a bit hard on the patient.

## Discussion

### Preliminary Findings

This study investigated health care professionals’ perspectives toward current follow-up care and the added value of an eHealth application monitoring QOL via patient-reported outcomes (PROs; Measure) followed by automatically generated tailored feedback (Learn), and personalized advice on supportive care (Act).

### Barriers in Referral to Supportive Care and Health Care Professionals’ Acceptance of an eHealth Application

The results of this study showed that current referral to optimal supportive care is limited due to several barriers, such as limited consultation time and a lack of overview of supportive care options. Our data support previous studies that have obtained insights into these barriers [7-9,22,23]. Furthermore, health care professionals clearly indicated they expected survivors to mention their symptoms. However, previous studies have shown that survivors themselves also experience barriers possibly resulting in unmet needs [8,10,14]: emotional barriers, such as not wanting to complain after surviving cancer, and practical barriers, such as not wanting to burden their physician. By automating the referral process to supportive care by means of an eHealth application, a barrier such as not wanting to burden their physician may be removed. In general, health care professionals expected that the proposed eHealth application could optimize the referral to supportive care.

### Content, System, and Service Quality of OncoKompas

Overall, health care professionals were most pleased with the service quality of the application but mentioned several considerations regarding its system and content quality.

Our study showed that health care professionals concluded that OncoKompas was useful for a limited group of (HNC) survivors. A frequently mentioned barrier was lack of Internet access, which is remarkable as a large majority of the Dutch population (90.4%) has access to the Internet; 80% of 65-75 year olds indicated they used the Internet [24]. Therefore, access to the Internet seems to have become less of a barrier and the emphasis should be on developing an application congruent with eHealth literacy skills of end users. The needs assessment among cancer survivors showed that they required the application to be easily comprehensible [14].

Health care professionals in our study underlined the importance of tailoring the application. In the needs assessment among cancer survivors, tailoring was also deemed important. Patients, however, mentioned doubts about the degree of tailoring that is possible [14]. According to health care professionals, a lack of tailoring could lead to a loss of interest, possibly leading to nonadherence [25]. Health care professionals suggested that only select topics of concern to a user should be provided to improve tailoring.

Considering the content quality of OncoKompas, the majority of issues mentioned were related to the use of PROs. Usage of PROs resulted in overlap between items (as individual items cannot be deleted from validated PROs). Additionally, health care professionals mentioned comprehensibility issues: they assessed several PROs as too difficult. Although we strived for readability at the 10^th^grade level in all texts in the “Learn” and “Act” components of OncoKompas, validated PROs are not always at this reading level.

This study demonstrated that most health care professionals expect that the application will support survivors in obtaining appropriate and timely supportive care tailored to their symptoms [14]. This is in line with results of the needs assessment among cancers survivors. They expected similar advantages in receiving information on supportive care options tailored to their specific needs, for example, the ability to find supportive care options on their own and to take actions toward their symptoms [14]. In directing the HNC survivor to optimal supportive care, OncoKompas meets the objective of the current cancer care navigation movement toward ensuring cancer survivors receive adequate follow-up and supportive care [26,27]. However, some health care professionals in our study doubted whether survivors would know what to do after completing OncoKompas. They expected that HNC survivors could get lost in the supportive care options they can choose from, possibly leading to a lack of action. Given the evidence that more options and choice equals more stress and less action [28], the number of supportive care options that OncoKompas offers to the participant is limited to 3 recommendations.

### Implementation of eHealth

Health care professionals differed in their opinion whether OncoKompas should be implemented as a self-management application or a *supported* self-management application. The consequences of implementation on existing working procedures were discussed in interviews with those who preferred to implement OncoKompas as a *supported* self-management application, for example, incorporating an alert system in the hospital patient information system. Other health care professionals were of the opinion that survivors are responsible for their own well-being and that because of the importance in empowering the survivor and respect for the survivor’s privacy, the application should be implemented as a stand-alone self-management instrument. Wiggers et al [29] reported that implementing a *supported* self-management eHealth application in routine clinical practice increases the complexity of existing working procedures, possibly leading to low uptake of an eHealth application. This barrier may be avoided when implementing the application as a self-management tool. Both options offer advantages in clinical practice: supported self-management applications may be more suitable for survivors who lack eHealth literacy skills, while other cancer survivors may be more empowered by a stand-alone self-management instrument. Consequences of both options need to be studied further.

### Strengths and Limitations

This study is limited due to the small number of health care professionals involved. Another limitation is that it might have been difficult for health care professionals to view an eHealth application from the survivors’ perspective. However, the use of a participatory design approach, including health care professionals from different academic hospitals as well as combining these results with cancer survivors’ perspectives [14], covered all main aspects. The added value of usability research is limited when weaknesses are mentioned that could have been prevented in the design process. Because there are no similar applications in oncology, the results of our study add value and can be used as a guide for designing other applications. A strength of this study is that we also gained insight into implementation requirements of eHealth in clinical practice.

### Conclusion

Health care professionals experienced a variety of barriers in the current organization of supportive cancer care, such as a lack of overview of options. Health care professionals expected that the use of an eHealth application that monitors QOL and provides automatically generated personalized advice and referral to supportive care options may be helpful in eliminating some of these barriers. However, they also highlighted some concerns. They mentioned that the application may not be useful for all HNC survivors due to limited eHealth literacy and an older age. Cognitive walkthroughs revealed several points for optimizing the prototype of the application, including improved tailoring. Health care professionals expected several advantages for survivors: insight into the interdependence of symptoms for cancer survivors, (earlier) referral to adequate supportive care, and increased patient empowerment. Finally, useful recommendations for developing an efficient implementation strategy appeared from the interviews. It can be concluded that including health care professionals in an early phase of a participatory design approach is valuable in designing an eHealth application and an implementation strategy that meets stakeholders’ needs.

## Appendix

Multimedia Appendix 1 System, content, and service quality OncoKompas (strengths and weaknesses).

Multimedia Appendix 2 Implementation of OncoKompas.

## Abbreviations

CW: cognitive walkthrough
HNC: head and neck cancer
PROs: patient reported outcomes
QOL: quality of life
