# Supplementary material for: An eHealth Application in Head and Neck Cancer Survivorship Care: Health Care Professionals' Perspectives
Source: J Med Internet Res. 2015 Oct 21;17(10):e235. doi: 10.2196/jmir.4870 (PMC4642398; doi:10.2196/jmir.4870)
Supplement: Multimedia Appendix 1 [file jmir_v17i10e235_app1.pdf]

System, content, and service quality OncoKompas (strengths and weaknesses).

	Strengths	Weaknesses
**System quality**		
	**Ease of access**<br>• Suitable for survivors: (1) that already take good care of themselves, (2) are well-educated, (3) younger people, (4) have a next of kin who can help<br>• Suitable for HNC population: (1) are retired, (2) are able to book vacations online, (3) new population: HPV virus, (4) eHealth may limit the impairments HNC survivors may face<br>• Available in between HCP consults<br>• 24 hours availability from the home situation	**Ease of access**<br>• Not suitable for survivors that: (1) do not speak Dutch or are illiterate, (2) are cognitive or visually impaired, (3) are elderly, (4) have no Internet access or a lack of eHealth literacy skills, (5) are not motivated, (6) do not take care of their well-being, (7) have few symptoms<br>• Not suitable for HNC population: (1) don't like to read, (2) low educational level<br>• The application is built in Flash and therefore not suitable for use on tablets
	**Ease of use**<br>• Clear navigation structure: simple lay-out, clear main menu	**Ease of use**<br>• Unclear navigation structure: interface too busy, too much scrolling required
	**Tailoring / customization**<br>• Possibility to skip the sexuality topic is positive<br>• Survivors can choose between supportive care options which is of added value	**Tailoring / customization**<br>• Survivor should decide which symptoms to monitor<br>• Information, advices provided is / are too general<br>• Unclear that advice is tailored to the specific survivor
	**Conditioning**<br>• Option to print the results to share these with the HCP<br>• Patients receive concrete, interesting and tailored advice: asset to surfing	**Conditioning**<br>• Lack of a trigger to return to the application<br>• Lack of a 'check' if the survivor has taken action<br>• System should provide reminders to take action
**Content Quality**		
	**Evidence-based**<br>• Use of validated PROs: reliable<br>• Evidence-based content, advices and supportive care options provided in line with HCPs' expectations	**Evidence-based**<br>• Tension between goal *OncoKompas* and usage of validated PROs
	**Comprehensibility**<br>• Clear questioning: comprehensible questions<br>• Information and supportive care options offered at different levels of understanding<br>• Advice easy to follow; when complicated accurate referral to HCP	**Comprehensibility**<br>• Complicated questioning: multi-interpretable, similar, difficult, inappropriate intonation and formulation<br>• Complicated answering categories<br>• Difficult and abstract terminology<br>• Difference unclear between advised and alternative supportive care options
	**Complete**<br>• Complete picture: all QOL aspects included<br>• In-depth questioning<br>• Visibility of interdependence of QOL domains<br>• Complete overview supportive care options<br>• Survivors can choose between different supportive care options	**Incomplete**<br>• Clear introduction to PROs is missing<br>• Too little focus on interdependence between domains<br>• No perspective offered about the "normal" population<br>• Missing (information on) supportive care options: eg regional options, whether you need a referral, costs, inclusion in a quality register, option to get in contact<br><br>**Over complete**<br>• Too much text to read when you are ill/recovering<br>• Information is provided twofold<br>• Including flyers: more information leads to more questions<br>• Too many supportive care options provided

Service Quality		
	**Perceived usefulness "identification of symptoms"**<br>• Symptoms may become more clear by means of PROs<br>• Leads to clarification of request for help	**Perceived lack of usefulness "identification of symptoms"**<br>• Lack of nuance in identifying symptoms<br>• Impossible to tailor system as much as a consultation
	**Perceived usefulness "informing participants"**<br>• Generates new insights and knowledge for survivors<br>• *Recognition:* validation of survivors feelings<br>• Approachable for mild symptoms and delicate topics, survivors do not want to burden their physician with	**Perceived lack of usefulness "informing participants"**<br>• Easier for survivors to contact outpatient clinic<br>• Possibly wrong information gathering by wrong clicks<br>• Difficult for already tired participants to act upon personalized advice
	**Perceived usefulness "referring participants"**<br>• More specific, earlier, easier referral to supportive care<br>• Survivor can take initiative in asking for a referral<br>• Referral to own physician supports survivors to discuss symptoms with their physician	**Perceived lack of usefulness "referring participants"**<br>• Leads to confusion when many advices are prompted<br>• During consultation problems are easier to prioritize<br>• Not clear for survivors what to do after finishing *OncoKompas*<br>• Success depends on the assertiveness of the survivor
	**Perceived usefulness "overall service"**<br>• Support in post-treatment period<br>• Increased patient empowerment<br>• Increased attention for QOL of survivors<br>• Improved preparation for consultation with physician	**Perceived lack of usefulness "overall service"**<br>• Increased workload HCPs<br>• Increased worries about symptoms that survivors were not aware of and an increased focus on cancer<br>• Symptoms discussed in *OncoKompas* not mentioned to HCPs
