# Supplementary material for: An eHealth Application in Head and Neck Cancer Survivorship Care: Health Care Professionals' Perspectives
Source: J Med Internet Res. 2015 Oct 21;17(10):e235. doi: 10.2196/jmir.4870 (PMC4642398; doi:10.2196/jmir.4870)
Supplement: Multimedia Appendix 2 [file jmir_v17i10e235_app2.pdf]

Implementation of OncoKompas.

**Procedure introducing OK to patient**	
Accurate introduction of OncoKompas<br>• According to a routine, to prevent that survivors that may benefit from *OncoKompas* are not referred	• Through (1) a flyer, (2) a website, (3) posters in waiting area, (4) a demo of *OncoKompas*<br>• Included in a care pathway (eg after dismissal)<br>• At set times/moments (eg after dismissal)<br>• Responsibility introduction with one person<br>• Availability of a trigger for introduction for HCPs so it cannot be forgotten
**Implementation as self-management application**	**Implementation as supported self-management application**
Stimulates survivor empowerment<br>• Survivors have to take action themselves<br>• Enables survivors to indicate priorities during consultation<br>• Survivor determines how to handle symptoms and not the physician<br>• Survivor needs to be motivated to address his complaints, otherwise stimulation by HCP will not be effective	*Responsibility well-being patients with HCP*<br>• Self-management not fully accepted in health care<br>• Responsibility well-being always (partially) with HCP<br>• HCP responsible when survivor receives suboptimal care<br>• QOL difficult to act upon by HCPs if they don't have insight into results<br>• Important that symptoms unrelated to a specific specialty (eg anxiety, fatigue) receive attention and are noticeable
Survivor is responsible for own well-being<br>• Responsibility lies with survivor to take action upon symptoms<br>• Referral by *OncoKompas* in case of suboptimal QOL relieves HCPs' responsibility<br>• Survivor can bring printed results to consultation	*Feedback through access OncoKompas or system alert*<br>• HCPs are interested in *OncoKompas* results of survivors<br>• Feedback creates opportunity to discuss results during consultation<br>• HCPs want an alert when survivor has a suboptimal QOL and does not take action<br>• Survivor without printer cannot take results to HCP
Privacy of survivor is protected<br>• Survivor is able to choose what to share with the HCP<br>• No social desirability because HCP cannot view results<br>• HCPs doubt whether survivors would like their results known by HCPs	*Problems surrounding privacy survivors*<br>• Survivors need to be well informed about who has access to their information and give consent<br>• Some aspects of *OncoKompas* are of no importance to HCPs (such as financial issues)<br>• IT issues (accessibility) in how *OncoKompas* would be safe to use for HCPs
Difficult to discuss results during regular consultation<br>• Time pressure<br>• Other priorities during consultation (cancer recurrence)<br>• Problems in prioritizing during consultation	*Requires action from HCP*<br>• Survivors expect HCP to be aware of their results<br>• Lack of HCPs' action may lead to disappointment and have a negative impact on doctor-patient relation<br>• Dependence of survivor in doctor-patient relationship requires action from HCPs
